# Elevation of CD40/CD40L Inflammatory Pathway Molecules in Carotid Plaques from Moderate-and-Severe Obstructive Sleep Apnea Patients

**DOI:** 10.3390/diagnostics11060935

**Published:** 2021-05-22

**Authors:** Ewa Migacz, Wioletta Olejarz, Alicja Głuszko, Katarzyna Bednarek-Rajewska, Robert Proczka, David F. Smith, Stacey L. Ishman, Wojciech Kukwa

**Affiliations:** 1 Department of Otorhinolaryngology, Faculty of Dental Medicine, Medical University of Warsaw, 02-097 Warsaw, Poland; ewa.migacz@gmail.com; 2 Department of Biochemistry and Pharmacogenomics, Faculty of Pharmacy, Medical University of Warsaw, 02-097 Warsaw, Poland; wioletta.olejarz@wum.edu.pl; 3 Centre for Preclinical Research, Medical University of Warsaw, 02-097 Warsaw, Poland; alicja.gluszko@wum.edu.pl; 4 Department of Biochemistry, Faculty of Medicine, Medical University of Warsaw, 02-097 Warsaw, Poland; 5 Department of Clinical Pathology, Poznan University of Medical Sciences, 61-701 Poznan, Poland; szlapus@icloud.com; 6 Cardiology Center Jozefow, American Heart of Poland, 05-410 Warsaw, Poland; ramjup@poczta.onet.pl; 7 Divisions of Pediatric Otolaryngology, Pulmonary Medicine, and the Sleep Center, Cincinnati Children’s Hospital Medical Center, Cincinnati, OH 45229, USA; David.Smith3@cchmc.org (D.F.S.); stacey.ishman@cchmc.org (S.L.I.); 8 The Center for Circadian Medicine, Cincinnati Children’s Hospital Medical Center, Cincinnati, OH 45229, USA; 9 Department of Otolaryngology-Head and Neck Surgery, University of Cincinnati School of Medicine, Cincinnati, OH 45229, USA

**Keywords:** obstructive sleep apnea, CD40/CD40L, MCP-1, MMP-9, inflammation, atherosclerosis

## Abstract

A chronic inflammatory process characteristic of obstructive sleep apnea promotes vascular endothelial dysfunction and atherogenesis. This process can lead to destabilization and rupture of cardiovascular plaques, which clinically manifests as an acute coronary syndrome or stroke. The aim of this study was to investigate the inflammatory pathway leading to plaque destabilization in non-to-mild and moderate-to-severe groups of OSA patients. This prospective study involved enrollment of patients scheduled for endarterectomy. A sleep study was performed prior to surgery. Immunohistochemistry was performed on atherosclerotic plaques from carotid arteries obtained during standard open endarterectomy to determine levels of CD40, CD40L receptors, MCP-1, and MMP-9. The 46 patients included 14 controls, 13 with mild, 11 with moderate, and 8 with severe OSA. Increased expression of CD40, CD40L receptors, MCP-1, and MMP-9 were found to be proportionate with OSA severity. However, significant differences among groups were observed only for MCP-1 (*p* = 0.014). Increased expression of inflammatory markers (CD40, CD40L, MCP-1, MMP-9) is associated with increasing OSA severity. This suggests the CD40-CD4-L inflammatory pathway may contribute to plaque instability and rupture in OSA patients.

## 1. Introduction

Obstructive sleep apnea [1] is one of the most common sleep disorders among adults. It is characterized by recurrent episodes of partial or complete upper airway collapse during sleep [2]. OSA has frequently been associated with atherosclerosis [3]. Systemic inflammation, oxidative stress, vascular smooth muscle cell activation, increased adhesion molecule expression, monocyte/lymphocyte activation, increased lipid loading in macrophages, lipid peroxidation, and endothelial dysfunction all lead to plaque formation [4]. Initially stable atherosclerotic plaques tend to transform to unstable plaques, but the mechanism of this transition is not fully understood [5]. An unstable plaque is vulnerable to rupture, which may cause acute cardiovascular episodes such as stroke or acute coronary thrombosis.

OSA is considered as a persistent, low-intensity, inflammatory state [6]. One of the important inflammatory pathways is driven by a cluster of differentiation 40-cluster of differentiation 40-ligand (CD40-CD40L) dyads. It enhances leukocyte recruitment to the sites of vascular inflammation. In advanced lesions, it stimulates macrophages to increase production of matrix metalloproteinases (MMP) [7], particularly MMP-9, which is responsible for plaque destabilization and rupture [8]. Monocyte chemoattractant protein -1 (MCP-1) is another protein that promotes plaque vulnerability. It is expressed in macrophage-rich areas bordering the necrotic lipid core. This monocyte chemoattractant protein is also present in the early stages of atherosclerotic lesion formation [9]. It is produced by endothelial cells, smooth muscle cells, and/or monocytes in response to CD40L [10]. Moreover, activation of the MCP-1/CCR2 pathway induces expression of MMPs, and thus enhances plaque destabilization.

CD40-CD40L pathways are involved in atherosclerotic plaque generation and destabilization by inducing the expression of cytokines, chemokines, growth factors, matrix metalloproteinases, and pro-coagulant factors [11,12]. The increased prevalence of vascular diseases in OSA is caused by hypercoagulable state. Treatment with CPAP decreased plasma fibrinogen levels, platelet activity, and activity of clotting factor VII [13]. Persistent activation platelets in OSA patients, especially in obese subjects, results in enhanced spontaneous aggregability and changes in cytokine production [14]. Repeated temporary dearth of NO in the tissues causes long-term complications such as hypertension, myocardial infarction, and stroke [15].

These pathways and activators are well-known contributors to plaque destabilization and rupture [16]. The aim of this study was to investigate these markers in carotid plaques from patients with non-to-mild and moderate-to-severe OSA.

## 2. Materials and Methods

### 2.1. Patients and Tissue Samples

This was a prospective study enrolling consecutive patients scheduled for open endarterectomy from March 2014 to July 2017. Patients with nonocclusive high-grade atherosclerotic stenosis measuring > 70% luminal narrowing and a history of ipsilateral stroke or transient ischemic attacks (TIA) were scheduled for the procedure, according to the North American Symptomatic Carotid Endarterectomy Trial (NASCET) and the European Carotid Surgery Trial (ECST) guidelines [17]. Exclusion criteria included: BMI > 35 kg/m^2^, smoking > 20 years, diabetes mellitus treatment > 5 years, and previous treatment for OSA. Inclusion criteria included: completion of endarterectomy, plaque sampling, and a preoperative sleep study. Atherosclerotic plaques were fixed in 4% neutral buffered formaldehyde solution.

### 2.2. Sleep Study

A diagnosis for OSA was established prior to surgery and was based on a Home Sleep Apnea Test (HSAT) using the WatchPAT™ (Itamar Medical, Caesarea, Israel) portable sleep apnea diagnostic system [18]. The WatchPAT system measures peripheral arterial tonometry, oximetry, heart rate, actigraphy, body position, and snoring. OSA severity was classified by the apnea hypopnea index (AHI) as: mild, AHI ≥ 5; moderate, AHI ≥ 15; severe, AHI ≥ 30. The group with an AHI < 5 events/hour was considered the control. 

### 2.3. Immunohistochemistry

Immunohistochemical staining was performed to qualitatively identify antigens in sections of formalin-fixed, paraffin-embedded tissue. Tissue section formalin-fixed and paraffin-embedded were stained with the use of immunohistochemical method. Sections were deparaffinized and hydrated, following by epitope retrieval induced by heating, with the use of Tris/EDTA-based buffer pH 9 (Novocastra REF119-CE, Leica Biosystems, Nulssloch, Germany) and citrate buffer pH 6 (Novocastra REF113-CE, Leica Biosystems, Nussloch, Germany) for MMP9, MCP-1, and CD40, CD40L antibodies’ detection, respectively.

Subsequently, after neutralizing endogenous peroxidase activity and protein binding, sections were incubated overnight at 4 °C with diluted antibodies as follows: anti-CD40 1:250 (ab224639), anti-CD40L 1:400 (ab 52750), anti-MMP9 1:100 (ab58803), and anti-MCP1 1:100 (ab9858), all from Abcam, Cambridge, USA. Following, the tissue sections were incubated with secondary antibody (Post Primary Block) and subsequently with Novolink Polymer, each for 30 min. The following sections were incubated with secondary antibody and polymer. To detect antibodies, samples were incubated with substrate/chromogen, 3,3′-diaminobenzidine (DAB), and counterstained with hematoxylin. The procedure of staining was performed with the use of a Polymer Detection System kit (Novocastra REF140, Leica Biosystems, Nussloch, Germany) containing Peroxidase Block, Protein Block, Post Primary Block, Novolink Polymer, Hematoxylin, DAB Chromogen, Novolink DAB Substrate Buffer [19,20]. Results were interpreted using a light microscope (Axio Observer Z1, Zeiss, Jena, Germany, https://www.zeiss.com/microscopy/int/products.html/, accessed on 22 May 2021) equipped with Axiovision 4.8 software (Zeiss Microimaging GmbH, Jena, Germany, https://carl-zeiss-vision-axiovision-viewer.software.informer.com/4.8/, accessed on 22 May 2021), and illumination system LUMEN200 (Prior Scientific, Ltd., Jena, Germany, www.prior.com, accessed on 22 May 2021).

### 2.4. Evaluation of Immunohistochemistry

Results were expressed as a percentage of positive cells in the field of view (FOV), evaluating five randomly chosen high power fields (HPF ×200 magnification). The number of positive cells was evaluated according to the following 4-tiered classification: 0, negative reaction (less than 10% of stained cells); 1, low expression (11% to 50% of cells stained positive); 2, intermediate expression (51% to 75% of cells stained positive); 3, high expression (over 75% of cells stained positive). Experimental groups were masked to the pathologist to eliminate bias.

### 2.5. Statistical Analysis 

The results are expressed as the mean ± standard deviation [13]. A Student’s-*t* test was used to evaluate the differences between the groups, which were considered statistically significant at *p* < 0.05. Statistical analyses were performed with SPSS 23.0 software (IBM, Armonk, NY, USA, available online: https://www.ibm.com/analytics/spss-statistics-software, accessed on 22 May 2021).

## 3. Results

### 3.1. Study Participants

Sixty-eight patients met the inclusion criteria and were enrolled in the study. Forty-six subjects completed the protocol. Mean age was 73.2 ± 8.7 years. The control group (*n* = 14) included patients with an AHI < 5 events/hour. There were 13 patients (28%) with mild, 11 (24%) with moderate, and 8 (18%) with severe OSA (Table 1, Table S1). Patients were divided into two groups: non-to-mild and moderate-to-severe OSA.

### 3.2. Immunohistochemistry

**Figure 1:**
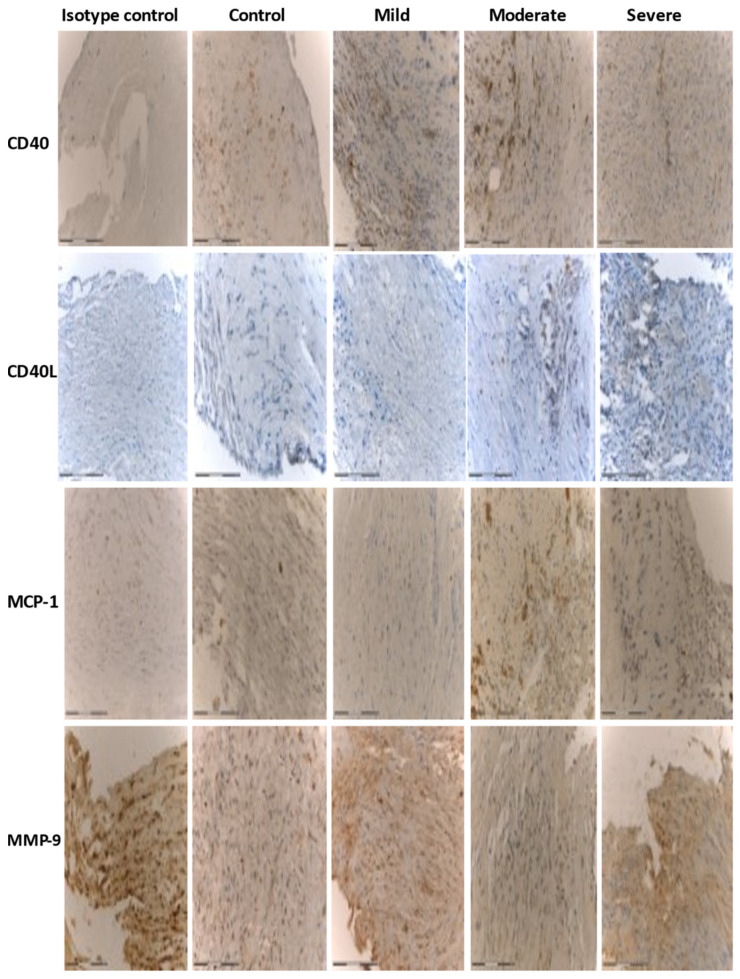
Immunohistochemical analysis of CD40, CD40L, MCP-1, and MMP-9 in carotid atherosclerotic plaque in patients undergoing carotid endarterectomy. Immunohistochemistry staining was performed with rabbit or mouse antibodies and with Isotype control IgG. Original magnification ×200. Scale bar = 200 µm.

**Figure 2:**
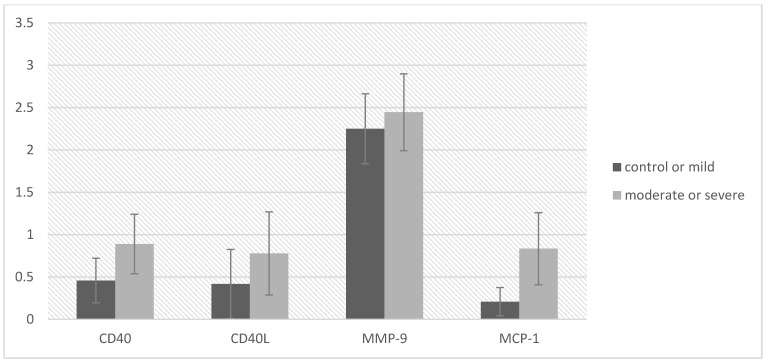
Levels of atherosclerosis markers in two groups of obstructive sleep apnea patients: control + mild and moderate + severe. Axis y shows the 4-tiered classification: 0, negative reaction (less than 10% of stained cells); 1, low expression (11% to 50% of cells stained positive); 2, intermediate expression (51% to 75% of cells stained positive); 3, high expression (over 75% of cells stained positive).

Immunohistochemistry demonstrated increased expression of CD40, CD40L, MMP-9, and MCP-1 in atherosclerotic plaques in the moderate/severe OSA group (Table 2, Table S2, Figure 1). Significant differences were observed for MCP-1 (*p* = 0.014) in patients with moderate-to-severe OSA, compared to the non-mild OSA group. Figure 2 shows that the expression of all markers increased with the severity of OSA. Most of the evaluated cells showed strong nuclear staining. In some cases, there was both strong nuclear and diffuse cytoplasmic staining (ranging from mild to strong diffuse cytoplasmic staining). The strongest immunohistochemical reaction was observed in stromal inflammatory cells: lymphocytes and macrophages, and in modified smooth muscle cells dispersed within the atherosclerotic plaque (especially within the shoulder, fibrous cap, and the core). 

## 4. Discussion

Cardiovascular diseases are clearly connected OSA. Intermittent hypoxia with reoxygenation, bursts of sympathetic activity, and exaggerated negative intrathoracic pressure are all pathophysiological sequelae of apneic events that, in turn, lead to cardiovascular consequences [21]. As many of these consequences seem to be due to progression of atherosclerosis, it is crucial to determine basic mechanisms of the OSA–atherosclerosis interplay. The present study demonstrated that moderate–severe OSA patients had increased levels of all measured markers of atherotic plaque vulnerability; however, statistically significant differences were only observed for MCP-1. CD40, CD40L, MMP-9, and MCP-1 were previously connected to plaque destabilization and rupture, along with many others [10]. Our study showed significant differences for MCP-1 (*p* = 0.014) and near-significant difference for CD40 (*p* = 0.056). Additionally, plasma levels of MCP-1 were previously shown to be elevated in OSA patients [22,23]. Chuang et al. demonstrated that gene expression of MCP-1 in peripheral blood monocytes was increased in patients with severe OSA [24]. Additionally, two studies analyzed the effect of OSA treatment on MCP-1 levels. A study by Perrini et al. documented that CPAP treatment reduced serum concentration of MCP-1, while the levels remained unaltered in the non-OSA and subtherapeutic groups (*p* < 0.05) [25]. The effect of adenotonsillectomy (T & A) was examined in OSA-positive obese children by Kheirandish-Gozal et al. [26]. They showed that effective OSA treatment resulted in significant reductions in MCP-1 serum levels (*p* < 0.003). The authors suggested that OSA treatment reduced the overall inflammatory state and resulting cardiovascular risk in those patients. This study also demonstrated levels of MMP-9 before and after the treatment, showing significant improvement (*p* < 0.0001). Vuralkan et al. showed a significant decrease in MMP-9 levels in adult patients after uvulopalatal flap (UPF) surgery (*p* < 0.05), but with no correlation to postoperative ODI values [27]. Multiple studies showed an increased level of MMP-9 in OSA patients [28,29,30,31]. Additionally, Wang et al. found that the MMP-9 level was positively correlated with OSA severity and consistent with the degree of hypoxia [32]. On the contrary, at least three studies showed no significant differences in MMP-9 values in OSA versus non-OSA subjects. One was performed in the paediatric population [33] and two others in adults [34,35]. The roles of MMPs in the inflammatory process associated with OSA were reviewed recently by two papers [36,37]. They have been associated with both oxidative stress and cardiovascular diseases by contributing to ischemia/reperfusion injury [37]. Wang et al. showed that MMP-9 is a risk factor for cardiovascular diseases in OSA patients by its involvement in the hypoxia-MMP-9-*β*2AR signalling axis [32]. Additionally, Hopps et al. confirmed higher plasma levels of MMP-9 and significant impairment of oxidative status in OSA patients [38]. Chuang et al. showed significantly higher mRNA expression of MMP-9 in monocytes in patients with severe OSA, which correlated to plasma MMP-9 levels [30]. MMP-9 has been associated with the progression of atherosclerosis and plaque rupture, and the development of vascular events in carotid atherosclerotic disease [39]. MMPs were expressed mainly at the shoulder region of the fibrous cap, which may promote weakening of the plaque following destabilization. In several studies, MMP levels were higher in acute coronary syndrome patients than in controls or patients with more advanced chronic heart failure [10].

Our study showed a slightly higher level of MMP-9 in moderate–severe OSA patients, but the results were not statistically significant. This may suggest that, in OSA patients, the plaque itself may not be the primary source of an increased level of plasma/serum MMP-9 levels, if these levels are truly increased in OSA patients. Our study showed that both CD40 and CD40L were increased in moderate/severe OSA patients, with near-statistical significance for CD40 (*p* = 0.056). As this is the first study that measures the levels of these markers in atherosclerotic plaques of OSA and non-OSA patients, there are no other data to compare in tissue samples.

Our study has limitations that should be discussed. First, the sample size was small, which could limit our statistical analyses. Second, we collected only plaque samples removed during open endarterectomy; therefore, the structure of the arterial wall could not be analyzed. Future studies should analyze plaque with a whole cross-section of the vessel wall. This will allow for a determination of the expression of the unstable plaque markers in the layers of the vessels. Finally, we did not obtain data regarding BMI in eight patients, which limited our ability to study the correlation of the inflammatory parameter expression and BMI.

## 5. Conclusions

The severity of OSA is associated with increased levels of all measured markers of atherosclerotic plaque vulnerability (CD40, CD40L, MCP-1, MMP-9), with significant differences for MCP-1 (*p* = 0.014) and near-significant difference for CD40 (*p* = 0.056). These data suggest that the CD40-CD4-L inflammatory pathway may contribute to plaque instability and rupture in OSA patients. Ours is consistent with previous studies which show that OSA carries an independent risk of acute coronary syndrome and stroke.

## Figures and Tables

**Table 1:** Patient demographic data and home sleep apnea test (HSAT) results in 4 groups of patients divided according to pAHI.

	Control and Mild	Moderate and Severe
Cases, *n*	27	19
Age, years	72.6 ± 7.65	73.9 ± 9.8
Female/Male, *n*	16/11	6/13
BMI, kg/m^2^	26.4 ± 2.75	30 ± 3.6 *
Neck circumference, cm	38 ± 3.9	40.8 ± 2.4 *
Waist circumference, cm	97.7 ± 8.9	105.6 ± 10.1 *
pAHI, events/h	6.3 ± 1.75	32.6 ± 8.2 **
ODI	2.4 ± 1.15	23.3 ± 6.9 **
ESS score	5.45 ± 4.8	8.3 ± 5.5 *

* *p* < 0.05, ** *p* < 0.0001 (Student’s *t* -test); BMI: body mass index; pAHI: peripheral arterial tone apnea/hypopnea index; ODI: oxygen desaturation index; ESS: Epworth sleepiness scale.

**Table 2:** Level of atherosclerosis markers in two groups of obstructive sleep apnea patients: control/mild and moderate/severe.

	Control and Mild OSA (*n* = 27)		Moderate and Severe OSA (*n* = 19)				95% CI		
	M	SD	M	SD	*t*	*p*	LL	UL	d Cohen
CD40	0.46	0.66	0.89	0.76	−1.97	0.056	−0.87	0.01	0.61
CD40L	0.42	1.02	0.78	1.06	−1.12	0.270	−1.01	0.29	0.35
MMP-9	2.25	1.03	2.44	0.98	−0.62	0.541	−0.83	0.44	0.19
MCP-1	0.21	0.41	0.83	0.92	−2.68	0.014	−1.11	−0.14	0.92

OSA—obstructive sleep apnea; M—mean; SD—standard deviation; *t*—Student’s t-test; *p*—significance value; CI—confidence interval; LL—lower limit; UL—upper limit.

## Data Availability

Data supporting reported results can be found at Department of Otorhinolaryngology, Faculty of Medicine and Dentistry, Medical University of Warsaw, Warsaw, Poland.

## Supplementary Materials

The following are available online at https://www.mdpi.com/article/10.3390/diagnostics11060935/s1, Table S1: Patient demographic data and home sleep apnea test (HSAT) results in 4 groups of patients divided according to pAHI, Table S2: Level of atherosclerosis markers in two groups of obstructive sleep apnea patients: control/mild and moderate/severe.

## References

[1] Zychowski K.E., Sanchez B., Pedrosa R.P., Lorenzi-Filho G., Drager L.F., Polotsky V.Y., Campen M.J. (2016). Serum from obstructive sleep apnea patients induces inflammatory responses in coronary artery endothelial cells. Atherosclerosis.

[2] Franklin K.A., Lindberg E. (2015). Obstructive sleep apnea is a common disorder in the population—A review on the epidemiology of sleep apnea. J. Thorac. Dis..

[3] Geovanini G.R., Wang R., Weng J., Jenny N.S., Shea S., Allison M., Libby P., Redline S. (2018). Association between Obstructive Sleep Apnea and Cardiovascular Risk Factors: Variation by Age, Sex, and Race. The Multi-Ethnic Study of Atherosclerosis. Ann. Am. Thorac. Soc..

[4] Drager L.F., Polotsky V.Y., Lorenzi-Filho G. (2011). Obstructive Sleep Apnea: An Emerging Risk Factor for Atherosclerosis. Chest.

[5] Virmani R., Kolodgie F.D., Burke A.P., Finn A.V., Gold H.K., Tulenko T.N., Wrenn S.P., Narula J. (2005). Atherosclerotic plaque progression and vulnerability to rupture: Angiogenesis as a source of intraplaque hemorrhage. Arterioscler. Thromb. Vasc. Biol..

[6] Kheirandish-Gozal L., Gozal D. (2019). Obstructive Sleep Apnea and Inflammation: Proof of Concept Based on Two Illustrative Cytokines. Int. J. Mol. Sci..

[7] Bosmans L.A., Bosch L., Kusters P.J., Lutgens E., Seijkens T.T. (2021). The CD40-CD40L Dyad as Immunotherapeutic Target in Cardiovascular Disease. J. Cardiovasc. Transl. Res..

[8] Johnson J.L. (2017). Metalloproteinases in atherosclerosis. Eur. J. Pharmacol..

[9] Bianconi V., Sahebkar A., Atkin S.L., Pirro M. (2018). The regulation and importance of monocyte chemoattractant protein-1. Curr. Opin. Hematol..

[10] Koenig W., Khuseyinova N. (2007). Biomarkers of Atherosclerotic Plaque Instability and Rupture. Arter. Thromb. Vasc. Biol..

[11] Zhang B., Wu T., Chen M., Zhou Y., Yi D., Guo R. (2013). The CD40/CD40L system: A new therapeutic target for disease. Immunol. Lett..

[12] Pamukcu B., Lip G.Y.H., Snezhitskiy V., Shantsila E. (2011). The CD40-CD40L system in cardiovascular disease. Ann. Med..

[13] von Känel R., Dimsdale J.E. (2003). Hemostatic alterations in patients with obstructive sleep apnea and the implications for cardiovascular disease. Chest.

[14] Gabryelska A., Łukasik Z.M., Makowska J.S., Białasiewicz P. (2018). Obstructive Sleep Apnea: From Intermittent Hypoxia to Cardiovascular Complications via Blood Platelets. Front. Neurol..

[15] Djupesland P.G., Haight J.S.J. (2003). Nitric Oxide (NO) and Obstructive Sleep Apnea (OSA). Sleep Breath..

[16] Viktorinova A. (2020). Potential clinical utility of macrophage colony-stimulating factor, monocyte chemotactic protein-1 and myeloperoxidase in predicting atherosclerotic plaque instability. Discov. Med..

[17] Saba L., Mallarini G. (2010). A comparison between NASCET and ECST methods in the study of carotids: Evaluation using Multi-Detector-Row CT angiography. Eur. J. Radiol..

[18] Zhang Z., Sowho M., Otvos T., Sperandio L.S., East R.J., Sgambati F., Schwartz A., Schneider H. (2020). A comparison of automated and manual sleep staging and respiratory event recognition in a portable sleep diagnostic device with in-lab sleep study. J. Clin. Sleep Med..

[19] Ramos-Vara J.A. (2017). Principles and Methods of Immunohistochemistry. Methods Mol. Biol..

[20] Magaki S., Hojat S.A., Wei B., So A., Yong W.H. (2019). An Introduction to the Performance of Immunohistochemistry. Methods Mol. Biol..

[21] Dempsey J.A., Veasey S.C., Morgan B.J., O’Donnell C.P. (2010). Pathophysiology of Sleep Apnea. Physiol. Rev..

[22] Ohga E., Tomita T., Wada H., Yamamoto H., Nagase T., Ouchi Y. (2003). Effects of obstructive sleep apnea on circulating ICAM-1, IL-8, and MCP-1. J. Appl. Physiol..

[23] Kim J., Lee C.H., Park C.S., Kim B.G., Kim S.W., Cho J.H. (2010). Plasma Levels of MCP-1 and Adiponectin in Obstructive Sleep Apnea Syndrome. Arch. Otolaryngol.-Head Neck Surg..

[24] Chuang L.-P., Chen N.-H., Lin Y., Ko W.-S., Pang J.-H.S. (2015). Increased MCP-1 gene expression in monocytes of severe OSA patients and under intermittent hypoxia. Sleep Breath..

[25] Perrini S., Cignarelli A., Quaranta V.N., Falcone V.A., Kounaki S., Porro S., Ciavarella A., Ficarella R., Barbaro M., Genchi V.A. (2017). Correction of intermittent hypoxia reduces inflammation in obese subjects with obstructive sleep apnea. JCI Insight.

[26] Kheirandish-Gozal L., Gileles-Hillel A., Alonso-Álvarez M.L., Peris E., Bhattacharjee R., Terán-Santos J., Duran-Cantolla J., Gozal D. (2015). Effects of adenotonsillectomy on plasma inflammatory biomarkers in obese children with obstructive sleep apnea: A community-based study. Int. J. Obes..

[27] Vuralkan E., Mutlu M., Firat I.H., Akaydin S., Sagit M., Akin I., Miser E., Ardic S. (2014). Changes in serum levels of MDA and MMP-9 after UPF in patients with OSAS. Eur. Arch. Oto-Rhino-Laryngol..

[28] Tazaki T., Minoguchi K., Yokoe T., Samson K.T.R., Minoguchi H., Tanaka A., Watanabe Y., Adachi M. (2004). Increased Levels and Activity of Matrix Metalloproteinase-9 in Obstructive Sleep Apnea Syndrome. Am. J. Respir. Crit. Care Med..

[29] Hopps E., Canino B., Montana M., Calandrino V., Urso C., Presti R.L., Caimi G. (2016). Gelatinases and their tissue inhibitors in a group of subjects with obstructive sleep apnea syndrome. Clin. Hemorheol. Microcirc..

[30] Chuang L.-P., Chen N.-H., Lin S.-W., Chang Y.-L., Chao I.-J., Pang J.-H.S. (2013). Increased matrix metalloproteinases-9 after sleep in plasma and in monocytes of obstructive sleep apnea patients. Life Sci..

[31] Feng X., Liu B., Wang J., Xiao X. (2011). Research on the serum levels of matrix metalloproteinase-9 and free fatty acids in OSAHS cases. J. Clin. Otorhinolaryngol. Head Neck Surg..

[32] Wang S., Li S., Wang B., Liu J., Tang Q. (2018). Matrix Metalloproteinase-9 Is a Predictive Factor for Systematic Hypertension and Heart Dysfunction in Patients with Obstructive Sleep Apnea Syndrome. BioMed Res. Int..

[33] Kaditis A.G., Alexopoulos E.I., Karathanasi A., Ntamagka G., Oikonomidi S., Kiropoulos T.S., Zintzaras E., Gourgoulianis K. (2010). Adiposity and low-grade systemic inflammation modulate matrix metalloproteinase-9 levels in Greek children with sleep apnea. Pediatr. Pulmonol..

[34] Bonanno A., Riccobono L., Bonsignore M.R., Bue A.L., Salvaggio A., Insalaco G., Marrone O. (2016). Relaxin in Obstructive Sleep Apnea: Relationship with Blood Pressure and Inflammatory Mediators. Respiration.

[35] Maeder M.T., Strobel W., Christ M., Todd J., Estis J., Wildi K., Thalmann G., Hilti J., Brutsche M., Twerenbold R. (2015). Comprehensive biomarker profiling in patients with obstructive sleep apnea. Clin. Biochem..

[36] Fang X., Chen J., Wang W., Feng G., Li X., Zhang X., Zhang Y., Zhang J., Xu Z., Tai J. (2020). Matrix metalloproteinase 9 (MMP9) level and MMP9-1562C>T in patients with obstructive sleep apnea: A systematic review and meta-analysis of case-control studies. Sleep Med..

[37] Franczak A., Bil-Lula I., Sawicki G., Fenton M., Ayas N., Skomro R. (2019). Matrix metalloproteinases as possible biomarkers of obstructive sleep apnea severity – A systematic review. Sleep Med. Rev..

[38] Hopps E., Presti R.L., Montana M., Canino B., Calandrino V., Caimi G. (2015). Analysis of the correlations between oxidative stress, gelatinases and their tissue inhibitors in the human subjects with obstructive sleep apnea syndrome. J. Physiol. Pharmacol. Off. J. Pol. Physiol. Soc..

[39] Heo S.H., Cho C.-H., Kim H.O., Jo Y.H., Yoon K.-S., Lee J.H., Park J.-C., Park K.C., Ahn T.-B., Chung K.C. (2011). Plaque Rupture is a Determinant of Vascular Events in Carotid Artery Atherosclerotic Disease: Involvement of Matrix Metalloproteinases 2 and 9. J. Clin. Neurol..

