# Supplementary material for: Elevation of CD40/CD40L Inflammatory Pathway Molecules in Carotid Plaques from Moderate-and-Severe Obstructive Sleep Apnea Patients
Source: Diagnostics (Basel). 2021 May 22;11(6):935. doi: 10.3390/diagnostics11060935 (PMC8224789; doi:10.3390/diagnostics11060935)
Supplement: Supplementary file 1 [file diagnostics-11-00935-s001.zip › diagnostics-1210786-supplementary.pdf]

diagnostics

MDPI

Supplementary Materials

Table S1. Patient demographic data and home sleep apnea test (HSAT) results in 4 groups of patients divided according to pAHI.

	Control	Mild	Moderate	Severe
Cases, n	14	13	11	8
Age, years	71.4 ± 8.1	73.8 ± 7.2	74.3 ± 11.2	73.4 ± 8.3
Female/Male, n	9/5	7/6	3/8	3/5
BMI, kg/m^2	26.0 ± 2.7	26.8 ± 2.8	28.6 ± 3.6	31.3 ± 3.5*
Neck circumference, cm	37.4 ± 3.9	38.6 ± 3.9	39.5 ± 2.2	42 ± 2.5*
Waist circumference, cm	97.7 ± 11.3	97.6 ± 6.5	100.3 ± 13.4	110.8 ± 6.8*
pAHI, events/h	3.6 ± 1.3	9.0 ± 2.2***	21.7 ± 3.1***	43.5 ± 13.3***
ODI	1.3 ± 0.6	3.5 ± 1.7***	13.5 ± 4.5***	33.1 ± 9.3***
ESS score	3.4 ± 1.5	7.5 ± 8.1	7.8 ± 4.8*	8.7 ± 6.2**

*$p < 0.05$, ** $p < 0.001$, *** $p < 0.0001$ versus control (Student's *t*-test); BMI: body mass index; pAHI: peripheral arterial tone apnea/hypopnea index; ODI: oxygen desaturation index; ESS: Epworth sleepiness scale. This table was previously published in Sleep and Breathing, Springer Journal, doi: 10.1007/s11325-020-02029-w. and is available by Creative Commons CC-BY license.

Table S2. Level of atherosclerosis markers in two groups of obstructive sleep apnea patients: control/mild and moderate/severe.

	**Control (n = 14)**		**Mild (n = 13)**		**Moderate (n = 11)**		**Severe (n = 8)**				
	M	SD	M	SD	M	SD	M	SD	F	*p*	η^2
MCP-1	0.18	0.40	0.23	0.44	0.70	0.95	1.00	0.93	2.71	0.070	0.20
CD40	0.45	0.69	0.46	0.66	0.80	0.79	1.00	0.76	1.35	0.273	0.10
MMP-9	2.27	1.01	2.23	1.09	2.30	1.16	2.63	0.74	0.27	0.845	0.02
CD40L	0.27	0.90	0.54	1.13	0.70	1.06	0.88	1.13	0.57	0.640	0.04

OSA—obstructive sleep apnea; M—mean; SD—standard deviation; *t*—Student's *t*-test; *p*—significance value;
